# Differences in cortical morphology and child internalizing or externalizing problems: Accounting for the co‐occurrence

**DOI:** 10.1002/jcv2.12114

**Published:** 2022-12-07

**Authors:** Yingzhe Zhang, Bing Xu, Hannah H. Kim, Ryan Muetzel, Scott W. Delaney, Henning Tiemeier

**Affiliations:** ^1^ Department of Epidemiology Harvard T.H. Chan School of Public Health Boston Massachusetts USA; ^2^ Department of Child and Adolescent Psychiatry/Psychology Erasmus MC University Medical Center Rotterdam The Netherlands; ^3^ Department of Social and Behavioral Sciences Harvard T.H. Chan School of Public Health Boston Massachusetts USA

**Keywords:** adolescence, comorbidity, externalizing disorder, internalizing disorder, neuroimaging

## Abstract

**Background:**

Childhood internalizing and externalizing problems frequently co‐occur. Many studies report neural correlates of either internalizing or externalizing problems, but few account for their co‐occurrence. We aimed to assess specific cortical substrates of these psychiatric problems.

**Methods:**

We used data from 9635 children aged 9–11 years in the baseline Adolescent Brain Cognitive Development Study. Internalizing and externalizing problem composite scales scores were derived from the Child Behavior Checklist. We standardized FreeSurfer‐derived volumes of 68 cortical regions. We examined internalizing and externalizing problems separately and jointly (covariate‐adjustment) in relation to cortical volumes, with and without adjusting for total brain volume (TBV) in multivariate linear regressions adjusted for demographics and multiple comparisons. We fit bifactor models to confirm the consistency of patterns exploring specific internalizing and specific externalizing problems. Sensitivity analyses included a vertex‐wide analysis and a replication in another large population‐based study.

**Results:**

In separate TBV‐unadjusted analyses, externalizing and internalizing problems were associated with smaller cortical volumes. If adjusted for externalizing behavior, however, larger cortical volumes were associated with internalizing problems, while smaller cortical volumes remained associated with externalizing problems after adjustment for internalizing problems. The bifactor model produced similar results, which were consistently replicated in another pre‐adolescent neuroimaging sample. These associations likely represent global effects: adjusting for TBV rendered most associations non‐significant. Vertex‐wise analyses confirmed global patterns.

**Conclusion:**

Our results suggest that internalizing and externalizing problems have globally opposing, and non‐specific associations with cortical morphology in childhood, which are only apparent if analyses account for their co‐occurrence.

## Graphical abstract

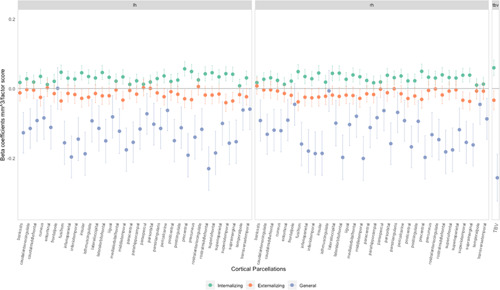

Many studies report neural correlates of either internalizing or externalizing problems, but few account for their co‐occurrence. In separate analyses in 9635 children aged 9–11 years from the Adolescent Brain Cognitive Development Study and 2365 children from the Generation *R* Study, externalizing and internalizing problems were associated with smaller cortical volumes. In mutually adjusted (internalizing and externalizing) and bivariate models, however, internalizing problems were associated with larger cortical volumes, while externalizing problems remained associated with smaller cortical volumes.

**Key points**

- Previous research has documented the frequent co‐occurrence of child internalizing and externalizing problems, but few neuroimaging studies accounted for this phenomenon.
- Our study revealed that internalizing and externalizing problems have global, non‐specific and directionally opposing associations with cortical volume, which were only detected after accounting for their co‐occurrence.
- Results emphasize the importance of accounting for the correlation between child internalizing and externalizing problems in neuroimaging studies. The opposite directions of associations of broad domains of internalizing and externalizing with cortical regions may help identify internalizing and externalizing problems.

## INTRODUCTION

Childhood mental health problems—including both internalizing (sadness, anxiety, depression, and somatic complaints) and externalizing (aggression and hyperactivity) problems—impose emotional and social burdens on families and society (Masten et al., 2005). Internalizing and externalizing problems frequently co‐occur in children with correlations ranging from 0.4 to 0.7 (Gilliom & Shaw, 2004), but the mechanism of the common co‐occurrence of internalizing and externalizing symptoms remains unclear. Researchers have hypothesized that underlying traits shaped by specific biological and environmental mechanisms may increase the vulnerability to psychological problems (Willner et al., 2016). This general vulnerability would contribute to the developmentally stable internalizing‐externalizing comorbidity. Alternatively, the presence of externalizing problems may be related to greater social problems that, in turn, increase the risk of developing emotional problems (McElroy et al., 2017). It has been argued that internalizing and externalizing symptoms should be studied together instead of being considered mutually exclusive, but doing so is uncommon, particularly in neuroimaging studies (Achenbach et al., 2016).

The frequent co‐occurrence of internalizing and externalizing problems suggests the presence of a general dimension of child psychopathology known as a “p‐factor” (Caspi & Moffitt, 2018). Bifactor models can be specified to construct a general psychopathology factor and “orthogonal” or specific factors (e.g., specific internalizing, externalizing, and attention factors) (Reise, 2012). Specifically, a general psychopathology factor was defined to underlie all problem items and the subscales items were specified to also load on one of the specific internalizing, externalizing, or attention factors (Caspi et al., 2014; Lahey et al., 2012). In a bifactor model, the overlap in symptoms or the non‐specific risk of internalizing, externalizing problems and attention problems can be removed.

Prior research related cortical morphology to internalizing or externalizing problems. A study using a bifactor model found that the common psychopathology factor was associated with smaller volumes in prefrontal areas, while the internalizing behavior‐specific factor was related to smaller insular volume (Snyder et al., 2017). Children with more externalizing symptoms had smaller frontal volumes (Weiland et al., 2014).

Currently, clinical diagnoses of anxiety or conduct problems—important subdomains of internalizing and externalizing problems—cannot rely on biomarkers since no physiological or brain imaging parameter is specific and reliable enough to aid assessment (Ogundele, 2018). Even though internalizing and externalizing problems do not formally define diagnoses in the Diagnostic and Statistical Manual of Mental Disorder, a neuroimaging study demonstrating specific brain morphological correlates of these symptom domains could help clinicians and researchers better understand common mental health problems.

Previous studies conducted in the Adolescent Brain Cognitive Development (ABCD) study have explored the associations between cortical volumes and child psychiatric problems. One study found that child cortical volumes were negatively associated with general psychopathology defined by a model using data from the Kiddie Schedule for Affective Disorders and Schizophrenia (Mewton et al., 2021). Another study reported similar negative associations between global cortical volumes and general psychopathology derived from exploratory factor analysis (EFA) of Child Behavior Checklist (CBCL) data (Durham et al., 2021). These studies focused on the general psychopathology and specific subscales using different factor structures and/or different statistical models, and neither study explored the structure of the empirical CBCL subdomains.

Against this background, we aimed to explore whether common internalizing and externalizing problems have specific brain volume substrates. We hypothesized that smaller insula volumes of internalizing symptoms become apparent only after adjusting for externalizing symptoms, and widespread smaller brain cortical volumes are associated with more externalizing symptoms. To test these hypotheses, we used data from pre‐adolescents on grey matter cortical volume and behavior problems from the ABCD study. We examined the associations between volumes in 68 cortical regions and internalizing and externalizing composite scale scores, separately, adjusted for each other, and additionally adjusted for total brain volume (TBV) with multivariate linear regression models. The latter adjustments were performed to investigate whether any specific regional association with psychiatric symptoms is indicative of global patterns. We conducted a complementary bifactor model analysis and vertex‐wise analysis to explore consistency across different analytical approaches. In addition, we replicated our analysis in an independent large population‐based cohort, the Generation *R* Study.

## METHODS

### Participants

We analyzed baseline data from the ABCD study (release 4.0) of children aged 9–11 years in the United States (Garavan et al., 2018). Researchers collected data on participant behavioral problems, brain morphology, demographic characteristics, and maternal psychopathology. Parents/caregivers provided written informed consent, and all children assented to a research protocol approved by the institutional review board at each data collection site (Clark et al., 2018). Of the 11,875 participants, we excluded 1088 subjects as one of the twins/triplets, 8 subjects with missing behavioral data, 828 subjects with missing cortical volume data, and 316 subjects with poor of brain images. This final analytic sample consisted of 9635 participants (81.1%).

### Child psychiatric symptoms

Child psychiatric problems were assessed at baseline using the primary caregiver's report of CBCL 6/18, a dimensional inventory of child behavioral problems (Achenbach & Rescorla, 2000). Caregivers answered on a three‐point frequency scale whether their child engaged in any of 119 specific problem behaviors in the past 6 months. Anxious/depressed, somatic complaints, and withdrawn/depressed scales were included as internalizing problem broadband domains (32 items), and aggressive behavior and rule‐breaking behavior scales were included as externalizing problem domains (35 items). The attention, social, and thought problem items were included in the bifactor model.

### Brain image acquisition and processing

Image data was collected at 21 sites across the U.S on 3T scanners from different vendors with resolution of 1.0 × 1.0 × 1.0 mm. The structural T1 images were corrected for gradient nonlinearity distortions with scanner‐specific and nonlinear transformations (Hagler et al., 2019). More information is in the supporting information. Cortical reconstruction and volumetric segmentation were performed using FreeSurfer v6.0.0. A combination of automated and manual methods was applied for quality control. Our study used cortical volume data mapped to 34 cortical regions of interest (ROIs) per hemisphere (68 total ROIs) based on the Desikan Killiany brain Atlas (Desikan et al., 2006) and TBV generated by FreeSurfer. We standardized volumes of all ROIs to mean 0 and variance 1.

### Covariates

Demographic factors were measured by parent/caregiver‐reported questionnaires. Age was assessed in months and sex as biological sex at birth. Race/ethnicity was categorized into the following: non‐Hispanic White, non‐Hispanic Black, Hispanic, Asian, Native American/Pacific Islander, and other/unknown. Race/ethnicity was considered as a social construct indicating social inequalities. Family annual income was categorized into 10 levels, from less than $5000 to more than $200,000. Parental education was evaluated with 21 categories (0 = ‘never attended school’, 21 = ‘doctoral degree’). Maternal psychiatric symptoms were evaluated with continuous sum score (range 0–240) of 120 questions from the Adult Self Report. We included study site and magnetic resonance imaging (MRI) scanner type as covariates despite harmonization of the processing procedure.

### Statistical analyses

The distribution of population characteristics is shown in Table 1. First, we examined the relation of each of the 68 standardized ROI volumes with continuous scores for internalizing and externalizing symptoms (modeled separately) using linear regression adjusting for all covariates. We adjusted the *p*‐values for multiple comparisons using false discovery rate (FDR, 68 regions) correction (Benjamini & Hochberg, 1995). Second, we repeated the modeling strategy but adjusted for broadband domains (i.e., we simultaneously included internalizing or externalizing problems in the model); again, we present FDR *p*‐values. Third, to examine global effects, we additionally adjusted for TBV, which is highly correlated with intracranial volume in children. Additionally, we ran separate models for boys and girls.

**TABLE 1:** Demographic factor and major outcome distribution of study sample

	Mean ± SD/*N* (%)
*N*	9635
Age (years)	9.9 ± 0.63
Sex	
Girls	4585 (47.6)
Boys	5050 (52.4)
Race	
non‐Hispanic white	5746 (59.7)
non‐Hispanic black	1487 (15.5)
Hispanic	2043 (21.2)
Asian	216 (2.2)
Native American/Pacific Islander	40 (0.4)
Other/Unknown	87 (0.9)
Family annual income	
Less than $50,000	2688 (27.9)
$50,000–$100,000	2497 (25.9)
More than $100,000	3618 (37.6)
Missingness	832 (8.6)
Highest parent education level (degree)	
High school graduation	1146 (11.9)
Bachelor's degree	5200 (54.0)
Graduate degree or above	3277 (34.0)
Missingness	12 (0.1)
Maternal psychopathology (point)	21.3 ± 18.1
Internalizing score (points)	5.1 ± 5.6
Externalizing score (points)	4.5 ± 5.9
Total CBCL score (points)	18.4 ± 18.1

Next, for exploratory analysis, we fit a bifactor model using data from all 119 CBCL items to delineate general psychopathology and specific domain latent factors. All symptoms loaded on a single factor representing general psychopathology, and the corresponding subscales were specified to load on specific externalizing, internalizing, or attention problems (Figure S1). Although the classic bifactor model generally assumes the specific factors to be orthogonal, this is not a fundamental requirement of the bifactor model (Park et al., 2011). In our bifactor model, we restricted the general psychopathology factor to be orthogonal with each specific factor and allowed the three specific factors to negatively correlate with each other. The model has all symptoms loading on a single factor representing general psychopathology, and the corresponding subscales were specified to load on specific externalizing, internalizing, or attention problems (Figure S1). Model fit was assessed using the root‐mean‐square error of approximation (RMSEA), the comparative fit index (CFI), and the Tucker–Lewis index (TLI). Root‐mean‐square error of approximation ≤0.05, and CFI and TLI ≥0.95 were interpreted as good model fit (Hu & Bentler, 1999). We computed factor scores for general behavior problems, and specific scores for internalizing, externalizing, and attention problems. In turn, we used these factor scores as outcomes in fully adjusted linear regression (with and without TBV adjustment) to further explore the associations between each of the 68 standardized ROI volumes and child psychiatric traits.

Next, we conducted fully adjusted vertex‐wise analyses of cortical volumes and (1) internalizing and externalizing problems additionally adjusted for each other and (2) the bifactor model. For the vertex‐wise analyses we used QDECR (Lamballais & Muetzel, 2021), a custom *R* package to perform multiple linear regression models at each cortical vertex. Correction for multiple testing was applied by using cluster‐wise corrections based on Monte Carlo simulations with a cluster forming threshold of 0.001, which yields false positive rates similar to full permutation testing (Greve & Fischl, 2018).

### Replication in Generation R

We attempted to replicate the results in an independent pediatric population‐based cohort, the Generation *R* Study. We included 2365 participants after excluding 608 children with missing behavioral assessment or cortical volumes, or as one of a sibling pair. Primary caregivers rated child behavior problems at a mean age of 10.1 (SD = 0.6) years using the CBCL 6/18. All covariate information was also collected by questionnaire. Child national origin was coded into 3 categories: Dutch, non‐Dutch western, and non‐western. Family monthly income was measured by 11 levels and recoded into 3 categories: low, medium, and high income. Maternal education was assessed with 6 categories (0 = ‘no education finished’, 5 = ‘Bachelor's degree or higher’). Maternal psychopathology was measured at 6 months postnatal with the Global Severity Index scores of the Brief Symptom Inventory. The MRI assessment details are in the supporting information.

We used the same analytical approach as above with the 68 regional neuroanatomical volumes and the CBCL internalizing and externalizing problem scores. Analyses were conducted separately for both broadband domains, jointly, and additionally adjusted for TBV. We also fit a bifactor model in Generation R. The extent of replication of the analyses using the Generation *R* Study was quantified by intraclass correlation coefficients (ICC) between estimates in different models from both studies across all ROIs per dependent variables.

Missing values in the covariates were imputed using the MICE package in R. All cortical parcellation abbreviations listed in the figures are presented in Table S1. All analyses were conducted with *R* statistical software version 4.0.3.

## RESULTS

### Sample characteristics

Demographic characteristics of the study population are displayed in Table 1 (mean [SD] age, 9.9 years [0.63 years]; 47.6% girls; 59.7% non‐Hispanic White race/ethnicity). 27.6% of families had an annual income less than $50,000; 54.0% of parents held a Bachelor's degree, and 34.0% of parents attained graduate education or higher. Mean internalizing and externalizing problem scores were 5.1 (SD = 5.6) and 4.5 (SD = 5.9), respectively. Internalizing and externalizing scores were moderately correlated (Pearson correlation 0.58). The differences between included and excluded samples are presented in Table S2: included samples were younger with a smaller proportion of non‐Hispanic White ethnicity, with less family income and more psychiatric problems. Additionally, higher family income and higher parent education were associated with less general psychopathology and specific externalizing problems but more specific internalizing problems (Table S3).

### Brain morphology and internalizing and externalizing symptoms

**FIGURE 1:**
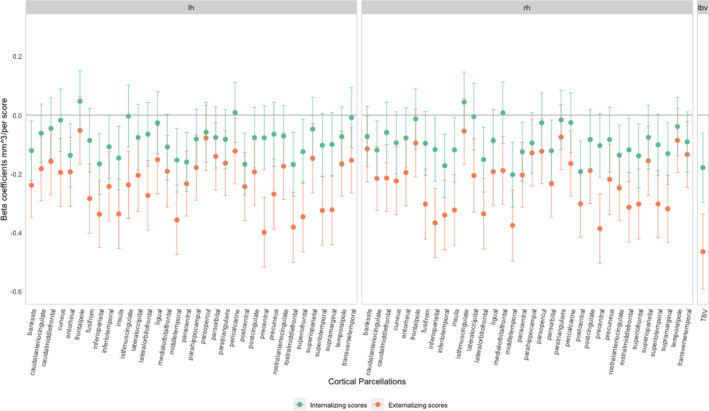
Associations between cortical volume and internalizing/externalizing problem scores adjusted for covariates in the Adolescent Brain Cognitive Development (ABCD) study. All associations were adjusted for age, sex, study sites, family income, highest parental education and maternal psychopathology.

In this results section, we will focus on patterns of associations. The direction of association between cortical volumes and internalizing symptoms (Figure 1) was negative in most regions as was that of TBV. Children with higher internalizing problem scores had, on average, smaller volumes in a few regions than children with fewer symptoms. These regions, including middle temporal and postcentral regions in both hemispheres, remained associated with internalizing problems after FDR correction. Similarly, cortical volumes were negatively associated with externalizing problems in all 68 regions, but most associations remained after FDR correction as did the association of the TBV with externalizing problems.

**FIGURE 2:**
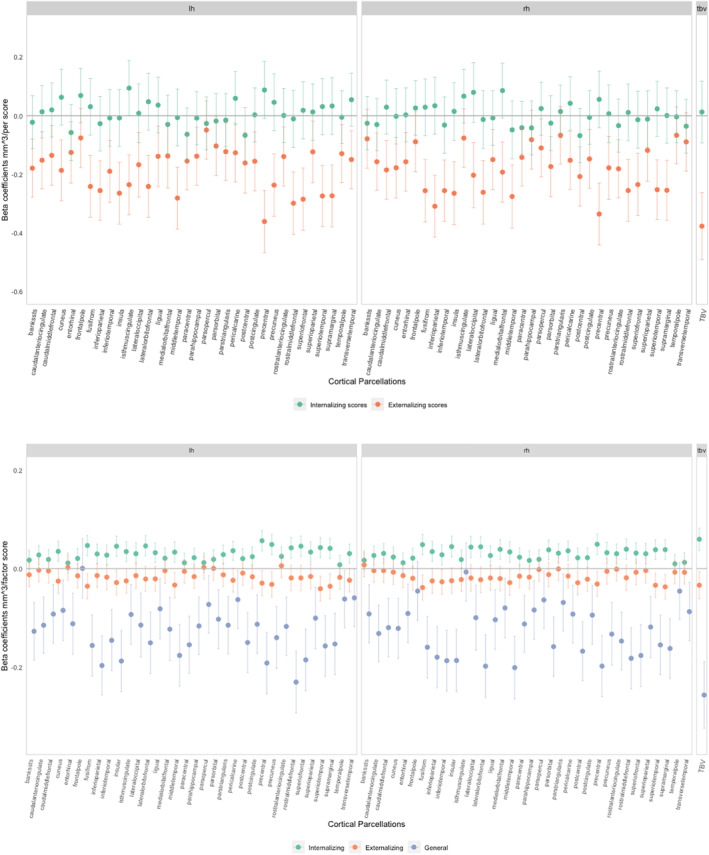
(A) Associations between cortical volume and internalizing/externalizing problem score adjusted for covariates and additionally adjusted for internalizing or externalizing symptoms in the Adolescent Brain Cognitive Development (ABCD) study; (B) Associations between cortical volumes and specific internalizing, specific externalizing and generalizing problem factors (bifactor model) in the Adolescent Brain Cognitive Development (ABCD) study. All associations were adjusted for age, sex, study sites, family income, highest parental education and maternal psychopathology.

When we examined the behavioral domains jointly (Figure 2A), we found a different pattern of point estimates for internalizing symptoms. The direction of associations between many cortical volumes and internalizing problem scores was now positive, although only one was nominally significant. A larger isthmus cingulate region of the left hemisphere was associated with higher internalizing scores but this did not survive FDR correction. In contrast, effect sizes of the negative associations between cortical volumes and externalizing problems adjusted for internalizing problems remained similar and were significant in most regions after FDR correction. When we adjusted these models for TBV (Figure S2A), we found no associations of any region with internalizing or externalizing problems. We found no differences in the patterns of effect estimates when stratifying our models by sex (Figures S3).

**FIGURE 3:**
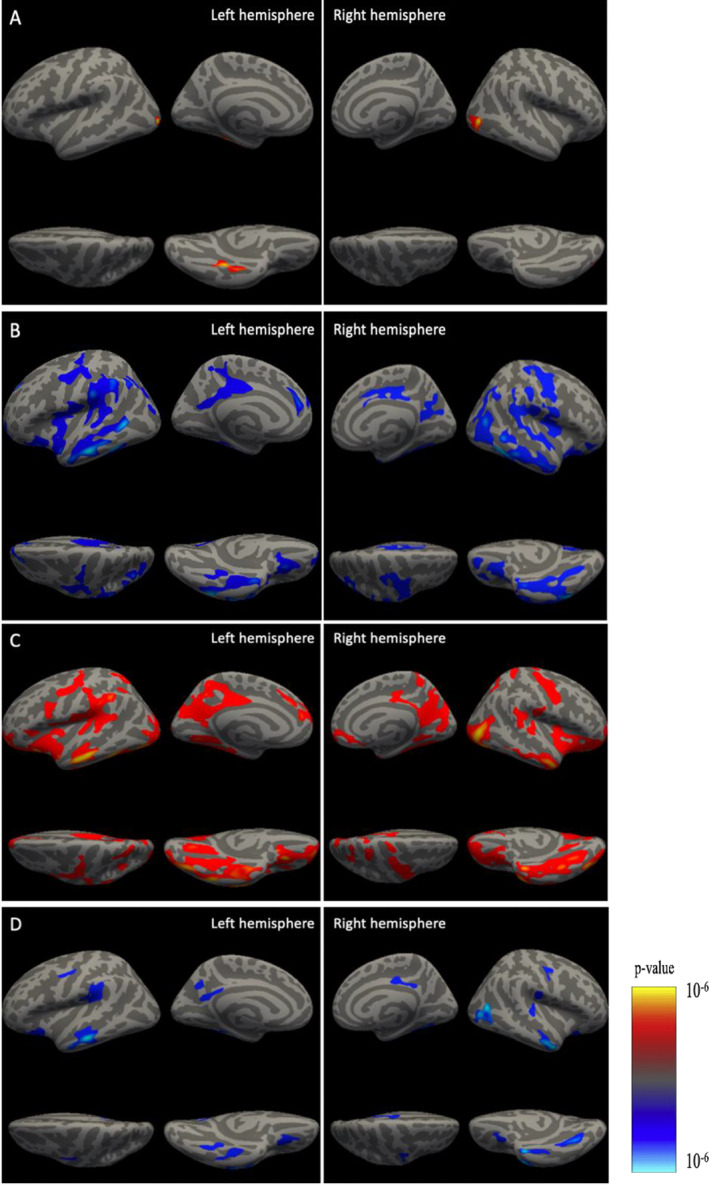
Vertex wise analysis of brain volume with both internalizing/externalizing problem score additionally adjusted for internalizing/externalizing problem score, and specific internalizing/externalizing factor score, all adjusting for covariates. (A) Internalizing problem score adjusted for covariates and externalizing problem score; (B) Externalizing problem score adjusted for covariates and internalizing problem score; (C) Specific internalizing factor adjusted for covariates; (D) Specific externalizing factor adjusted for covariates.

**FIGURE 4:**
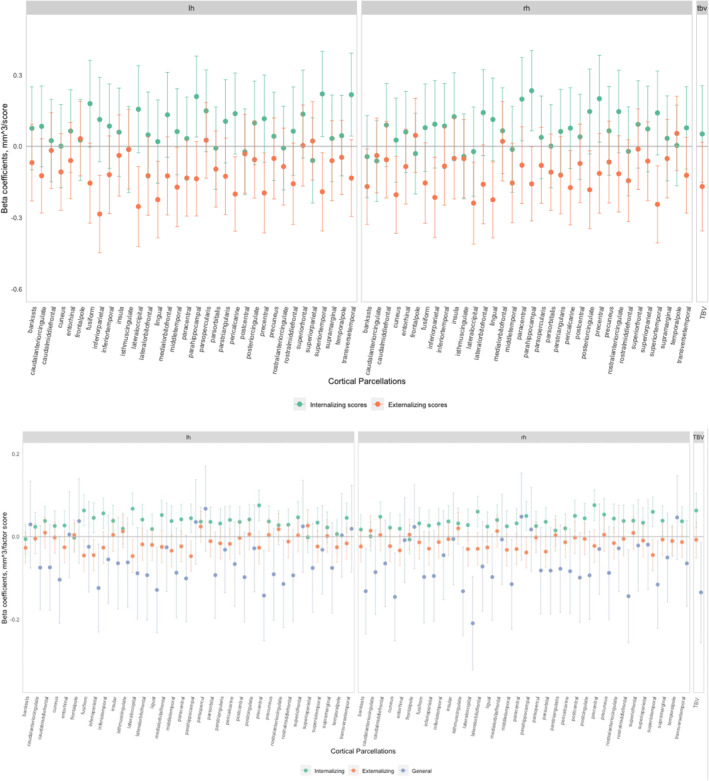
(A) Associations between cortical volume and internalizing/externalizing problem score adjusted for covariates and additionally adjusted for internalizing or externalizing symptoms in the Generation R Study; (B) Associations between cortical volumes and specific internalizing, specific externalizing and generalizing problem factor (bifactor model) in the Generation R Study. All associations were adjusted for age, sex, study sites, family income, highest parental education and maternal psychopathology. The model fit for the bifactor model in the Generation R study is excellent (comparative fit index (CFI) = 0.992, Tucker–Lewis index (TLI) = 0.980, root‐mean‐square error of approximation (RMSEA) = 0.048 (90% confidence interval = 0.038, 0.059)).

### Bifactor model

We constructed a bifactor model (Figure S1) with good model fit (CFI = 0.995, TLI = 0.988, RMSEA = 0.043 (90% confidence interval = 0.038, 0.048)). In the bifactor model, the general psychopathology factor was not related to the specific internalizing, externalizing or attention factor. However, the specific internalizing and externalizing factor were negatively correlated (*r* = −0.67).

Figure 2B shows the results of the fully adjusted linear regression models using the bifactor model loading scores. Children with larger cortical volumes in any cortical region had higher specific internalizing factor scores, and these estimates were significant in many regions after FDR correction. In contrast, most cortical volumes showed negative associations with specific externalizing problem scores but after FDR correction none remained. Likewise, all cortical parcellations except for the left frontal pole region showed negative relationships with the general psychopathology factor; most associations remaining significant after FDR correction. Hence, results from the bifactor model largely confirmed the pattern found in the analyses adjusting for internalizing or externalizing problems. However, in the bifactor model, the inversed pattern of associations of cortical regions with internalizing and externalizing problems was more obvious. Moreover, children with higher specific attention problem loading scores had, on average, smaller brain volumes (Figure S4).

We found no association between cortical volume in any ROI and specific internalizing or externalizing factor scores after adjusting for TBV (Figure S2B). Similarly, associations between cortical volumes and the general factor were attenuated by TBV adjustment; only a positive association of the right isthmus cingulate remained after FDR correction.

### Vertex‐wise analysis

Vertex‐wise analyses using both CBCL broadband domain scores and bifactor loading scores revealed consistent global cortical volume associations that mirror the regional analyses. In models using CBCL broadband domain scores, children with larger cortical volumes in several cortical clusters had more internalizing symptoms after adjusting for externalizing symptoms and other covariates. Specifically, higher internalizing problem scores were associated with larger volume in 3 cortical clusters in the left hemisphere (lateral occipital, fusiform, inferior temporal regions) and the lateral occipital region in the right hemisphere (Figure 3A). In contrast, children with smaller lower cortical volumes in several large clusters, including supramarginal, superior frontal, lateral orbitofrontal, precentral and postcentral regions in both hemispheres, and inferior parietal, rostral middle frontal in the left hemisphere (Figure 3B) had higher externalizing problem scores.

Vertex‐wise models using bifactor loading scores yielded consistent result patterns. Specific internalizing problems derived from the bifactor model loading scores suggested widespread positive associations across the brain (Figure 3C). For example, children with larger volumes of superior frontal, superior parietal and precentral regions were more likely to have internalizing problems. In contrast, children with smaller cortical volumes (supramarginal, middle temporal, and lateral orbitofrontal clusters in the left hemisphere, and precentral, fusiform, superior temporal, and middle temporal regions in the right hemisphere) had more specific externalizing problems after adjusting for all covariates (Figure 3D). Top clusters are listed in Table S4.

### Replication results in Generation R

Demographic characteristics of the study population are displayed in Table S5 (mean [SD] age, 10.11 years [0.57 years]; 51.0% girls; 64.7% Dutch race/ethnicity; 26.6% with monthly income less than €2000; 59.0% of mothers held a Bachelor's degree or higher. Internalizing scores and externalizing score were moderately correlated (*r* = 0.54).

In separate models (Figure S5), we observed that the directions of associations between many cortical regions and internalizing problem scores were negative. None of them survived FDR correction. Similar to the ABCD study results, smaller cortical volumes in several regions were related to more externalizing problem scores. After FDR correction, an association remained in 3 regions (inferior parietal and lingual regions in the left hemisphere and lateral occipital region in the right hemisphere).

We observed positive associations between many cortical volumes and internalizing problems when adjusting for externalizing problems (Figure 4A). As in the ABCD study, none of them survived FDR correction. In contrast, children with smaller cortical volumes of most regions had more externalizing problems when adjusted for internalizing problems. The inferior parietal region in the left hemisphere remained associated with externalizing problems after FDR correction. Lastly, in TBV adjusted models (Figure S6A), all associations between cortical volumes and internalizing or externalizing problems were strongly attenuated and only the inferior parietal region in the left hemisphere remained associated with externalizing problems.

The same bifactor model was applied to Generation *R* with a good model fit (see Figure 4B). Children with larger cortical volumes in most regions had higher specific internalizing factor scores; several regions remained positively associated with internalizing problems after FDR correction. Conversely, the associations between many cortical volumes and specific externalizing problems were negative, but none survived FDR correction. Therefore, results are largely consistent with the patterns observed in the internalizing or externalizing adjusted analyses in ABCD.

The range of ICC between the estimates from both studies was between 0.881 and 0.995 (Table S6) and can be considered as good evidence for replication. However, the directions of some associations (e.g., precuneus and supramarginal of the left hemisphere with internalizing problems) differed between the two samples.

## DISCUSSION

This large population‐based imaging study of common child psychiatric symptoms yielded two main findings that were consistent across analytical approaches. First, larger brain cortical volumes, although mostly not statistically significant, were associated with more internalizing symptoms only if externalizing problems were accounted for. In contrast, smaller volumes in most cortical regions were associated with more externalizing problems. Second, adjusting models for TBV rendered most associations non‐significant, which strongly suggests no specific associations underlie this pattern. Vertex‐wise analyses demonstrated consistent associations with previous models. The results were replicated with Generation *R* data with similar patterns of association of cortical volumes with internalizing and externalizing problems, although standard deviations of the effect estimates were larger in this smaller sample.

Few neuroimaging studies have investigated internalizing and externalizing traits jointly despite the substantial level of co‐occurrence between the two traits. Our analyses attempted to account for the co‐occurrence of internalizing and externalizing problems by two approaches. One approach was treating internalizing or externalizing as confounding factors for each other, with the assumption that one might precede the other and possibly the brain morphological changes. However, such adjustments may also be warranted if the assessments of internalizing or externalizing problems are impacted by misclassification. The other one was a more theory‐driven approach delineating specific internalizing and externalizing problems, and general psychopathology problems in a bifactor model. Although it is difficult to interpret subscales within a bifactor model, and such subscales are less generalizable, we found a clear contrast in the associations of brain volumes with specific internalizing and externalizing problems.

A key finding of our study is the widespread associations of cortical volumes with internalizing, externalizing, and general psychopathology problems in both ABCD and Generation R. Most specific associations disappeared with TBV adjustment. Previously, smaller gray matter volume in many cortical regions had been described in relation to the general psychopathology factor in both youths and adults, a global pattern that is reproduced in the current study (Kaczkurkin et al., 2019). Such global patterns can be best explained by a genetic vulnerability driven by multiple variants. There is good evidence that many genetic variants impact brain development broadly, for example, by affecting fundamental regulatory or physiological mechanisms (Parenti et al., 2020). Also, specific regional brain effects may be hard to detect if many different combinations of genetic variants with different effects are at play for internalizing or externalizing problems in the population. Similarly, early life environmental exposures, such as intrauterine stress or poor nutrition, may have a broad impact on cortical development. Further, very poor‐quality MRI images may result in such phenomena, but our analyses excluded children with less optimal image quality (Alfaro‐Almagro et al., 2021). Based on the widespread associations between multiple cortical regions and internalizing and externalizing problems, our study suggests that no specific regional biomarker differentiating internalizing and externalizing problems exists. Given the broad comorbidity observed in clinical practice, this is likely, to some extent, also applicable to the common and heterogeneous disorders presenting in clinical settings such as attention deficit hyperactivity disorder (ADHD), conduct disorder, or anxiety.

Importantly, we found that while internalizing problems were associated with larger cortical volumes, externalizing problems were associated with smaller volumes in both the ABCD and Generation *R* studies. These directionally opposing associations could be the result of a suppression effect caused by the correlation between internalizing and externalizing problems in our study samples (Owens et al., 2021). It is possible that the statistical removal of a confounding effect could increase the magnitude of the relationship between the exposure and outcome. Such a change would indicate suppression. Hence, in both multivariate regression and bifactor models, when externalizing scores were included as an additional predictor, the predictive power of cortical volumes for internalizing problems may have increased due to a suppression effect (Watson et al., 2013). These suppression effects of externalizing problems likely brought into focus opposing elements that are inherent—but largely hidden—in the overall score of internalizing problems and thus reversed the direction of the cortex‐internalizing problem association. Many homogeneous psychiatric symptom scales may actually contain components with distinct, at times antagonistic, neurobiological properties. For example, items such as mood change and sulking included in the aggression scale domains (age 6–18 years version) can capture symptoms also common to internalizing problems (sulking is in the internalizing domain in the CBCL 1.5–5 years version). On average, children with a general psychiatric vulnerability expressing both externalizing and internalizing problems have slightly smaller cortical brain volumes. As aggression has consistently been related to smaller cortical volumes, controlling for aggression in multivariate or bifactor models, may unravel the underlying negative association between brain volumes and those symptoms of the internalizing problems less comorbid with aggression or not driven by overlapping items.

Likely, the opposite directions of associations are influenced by both environmental and genetic effects. Childhood internalizing symptoms share some genetic vulnerabilities with externalizing psychiatric traits or disorders, such as ADHD, autism, and aggression. Genetic factors specific to internalizing and externalizing problems are likely but not known (Jami et al., 2020). Whether these specific genetic factors have a particular impact on the development of brain structures underlying the differential direction of association of cortical volumes can only be speculated (Lahey et al., 2009). The complex genetic, environmental, and developmental interplay in child psychopathology makes it difficult to identify the mechanisms underlying brain structural changes.

Our results with internalizing problems were different from a prior study showing no significant effects for the internalizing specific factor (Durham et al., 2021), likely due to different bifactor model specifications. In our model, we applied the default CBCL subscales, as this structure showed excellent model fit in our samples. An EFA is not necessarily better than the default psychometric structure as the results of an EFA largely depends on the sample. Furthermore, we allowed the specific factors to be correlated with each other.

Our results of global associations in different directions suggest that imaging might help distinguish between a morphological substrate that is more likely to underlie an internalizing or an externalizing problem in children with a psychiatric disorder in clinical practice. As these disorders have relatively distinct clinical treatments, such information may be meaningful. However, this remains speculative, and future studies, in particular longitudinal research, must show if brain morphology differentially predicts externalizing or internalizing symptoms.

Results from our vertex‐wise analyses were consistent with findings from our other models in both ABCD and Generation *R* studies, insofar as they suggested associations between behavioral problems and gray matter volume broadly across the cortex. The largest internalizing problem‐related cluster based on the vertex‐wise analysis is located in the lateral occipital region. Prior research suggests it plays a central role in object recognition (Grill‐Spector et al., 2001); differences in its volume have also been associated with adolescent depression (Schmaal et al., 2017). For externalizing problems, lateral orbitofrontal and supramarginal regions were the most strongly related regions. The lateral orbitofrontal region has been associated with aggression and impulsivity in psychiatric patients (Antonucci et al., 2006). However, our results must be interpreted cautiously as vertex‐wise analyses only highlight cortical clusters that exceed a pre‐set cut‐off for statistical significance. This makes it difficult to discern an overall pattern of associations between cortical structures and internalizing/externalizing problems, particularly if effect estimates in some regions may not be strictly statistically significant.

Prior research suggests that girls generally have a higher prevalence of internalizing problems, while boys present more externalizing problems (Martel, 2013). Whether neurostructural characteristics underlying these differences remain poorly understood. In our study, we found no sex‐specific differences in associations between brain volumes and common child psychiatric symptoms. These results may be due to the nature of a population‐based study, in which sex differences may be less prominent than in clinical settings (Biederman et al., 2002).

Our study has some limitations. First, internalizing and externalizing symptoms were assessed by parent/caregiver of the children. At age 9–11, children are less able to report on the whole spectrum of psychopathology than parent/caregiver. Even though ABCD collected youth brief problem monitor information, the instrument only contains 19 questions, whereas the CBCL is a well‐validated questionnaire with much broader scales for assessing child behaviors. Second, the difference between included and excluded samples might introduce selection bias. Yet, about half of the excluded samples were twins/triplets and the oversampling of twins/triplets in the ABCD study could also introduce selection bias. For example, non‐Hispanic White families and families with higher income were more likely to have twins/triplets. Last, we concentrated our analyses on cortical volume, since volumetric characteristics are the most commonly used ROIs measures.

In summary, larger cortical volumes were generally associated with more internalizing problems, particularly in the bifactor analyses when externalizing problems were accounted for. This may indicate a suppression effect: externalizing problems may mask the actual association between internalizing problems and brain characteristic. Smaller cortical volumes were generally associated with more externalizing problems in all analyses. These associations patterns were widespread, and largely not regionally specific. Our results were consistent across populations and independent of analytic methods. They demonstrate the importance of accounting for the co‐occurrence of internalizing and externalizing symptoms in neuroimaging research, and the potential for utilizing directions in associations with brain structures to help identify internalizing and externalizing problems.

## AUTHOR CONTRIBUTIONS

**Yingzhe Zhang**: Conceptualization; Formal analysis; Methodology; Project administration; Writing – original draft. **Bing Xu**: Formal analysis; Validation; Writing – review & editing. **Hannah H. Kim**: Methodology; Software; Visualization; Writing – original draft. **Ryan Muetzel**: Conceptualization; Data curation; Methodology; Writing – review & editing. **Scott W. Delaney**: Methodology; Supervision; Writing – original draft. **Henning Tiemeier**: Conceptualization; Data curation; Funding acquisition; Methodology; Project administration; Supervision; Writing – original draft.

## CONFLICTS OF INTEREST

Henning Tiemeier serves on the JCPP *Advances* Editorial Advisory Board. The remaining authors have declared that they have no competing or potential conflicts of interest.

## ETHICAL CONSIDERATIONS

The ABCD study was performed in line with the principles of the Declaration of Helsinki. The University of California at San Diego (San Diego, CA, USA) Institutional Review Board was responsible for the ethical oversight of the ABCD study. Parents/caregivers and children completed written informed consent and written assent, respectively. The secondary analysis of the data was approved by the Research Ethics Committee for the Harvard University (DAT21‐0705). The general design, all research aims and the specific measurements in the Generation *R* Study have been approved by the Medical Ethical Committee of Erasmus MC, University Medical Center Rotterdam. Participants need to give written informed consent for each phase of the study (fetal, preschool, childhood and adolescence period). From the age of 12 years onwards, children must sign their own consent form, in accordance with Dutch Law. At the start of each phase, children and their parents receive written and oral information about the study. Even with consent, when the child or the parents are not willing to participate actively, specific measurements are skipped or no measurements at all are performed.

## Supporting information

Supplementary Information S1

## Data Availability

Adolescent Brain Cognitive Development is an open‐access database and can be accessed at https://abcdstudy.org, held in the NIMH Data Archive. Data can be accessed following a data request to the NIH data access committee (https://nda.nih.gov/), which should include information on the planned topic of study. The following data sets were generated under NIMH Data Archive: Differences in cortical morphology and child internalizing or externalizing problems: accounting for the co‐occurrence #1862 (https://doi.org/10.15154/1528316). Generation *R* data can be requested by Email and proposal to the management team, mailing: datamanagementgenr@erasmusmc.nl or v.jaddoe@erasmusmc.nl.
