# Supplementary material for: Differences in cortical morphology and child internalizing or externalizing problems: Accounting for the co‐occurrence
Source: JCPP Adv. 2022 Dec 7;2(4):e12114. doi: 10.1002/jcv2.12114 (PMC10242825; doi:10.1002/jcv2.12114)
Supplement: Supplementary file 1 — Supplementary Information S1 [file JCV2-2-e12114-s001.docx]

**Supplementary Text on structural MRI preprocessing**

Image data was collected at 22 sites across the U.S on 3T scanners from different vendors (Siemens Prisma and Prisma Fit, General Electric MR 750, Philips Achievad Stream and Ingenia) with resolution as 1.0 × 1.0 × 1.0 mm. T1w and T2w structural images are corrected for gradient nonlinearity distortions using scanner-specific, nonlinear transformations provided by MRI scanner manufacturers (Jovicich et al., 2006; Wald et al., 2001). T2w images are registered to T1w images using mutual information (Wells et al., 1996) after coarse, rigid-body pre-alignment via within-modality registration to atlas brains. MR images are typically degraded by a smooth, spatially varying artifact (receive coil bias) that results in inconsistent intensity variations. Standard correction methods, such as those used by FreeSurfer (Dale et al., 1999; Fischl, 2012; Sled et al., 1998) are limited when compensating for steep spatial intensity variation, leading to inaccurate brain segmentation or cortical surface reconstruction. For example, brain tissue farther from the coils, such as the temporal and frontal poles, typically has lower intensity values, causing focal underestimation of the white matter surface, or even resulting in elimination of large pieces of cortex from the cortical surface reconstruction. Furthermore, brain tissue close to coils with extremely high intensity values may be mistaken for non-brain tissue (e.g., scalp).

In the Generation R study, 3959 children completed a complete T1-weighted sequence and 3687 received a T2 scan in the second wave at their age of 10-year-old. A total of 3937 scans were successfully reconstructed using FreeSurfer. More details of the MRI assessment in the Generation R study were described in a previous paper (White et al., 2018).

**Reference**

Dale AM, Fischl B, Sereno MI, 1999. Cortical surface-based analysis. I. Segmentation and surface reconstruction. Neuroimage 9, 179–194.

Fischl B, 2012. FreeSurfer. Neuroimage 62, 774–781.

Jovicich J, Czanner S, Greve D, Haley E, van der Kouwe A, Gollub R, Kennedy D, Schmitt F, Brown G, Macfall J, Fischl B, Dale A, 2006. Reliability in multi-site structural MRI studies: effects of gradient non-linearity correction on phantom and human data. Neuroimage 30, 436–443.

Wald L, Schmitt F, Dale A, 2001. Systematic spatial distortion in MRI due to gradient nonlinearities. Neuroimage 13, 50.

Wells WM 3rd, Viola P, Atsumi H, Nakajima S, Kikinis R, 1996. Multi-modal volume registration by maximization of mutual information. Med Image Anal 1, 35–51.

Sled JG, Zijdenbos AP, Evans AC, 1998. A nonparametric method for automatic correction of intensity nonuniformity in MRI data. IEEE Trans Med Imaging 17, 87–97.

White, T., Muetzel, R. L., El Marroun, H., Blanken, L. M. E., Jansen, P., Bolhuis, K., . . . Tiemeier, H. (2018). Paediatric population neuroimaging and the Generation R Study: the second wave. European Journal of Epidemiology, 33(1), 99-125. doi:10.1007/s10654-017-0319-y

**Figure S1: Bifactor model**


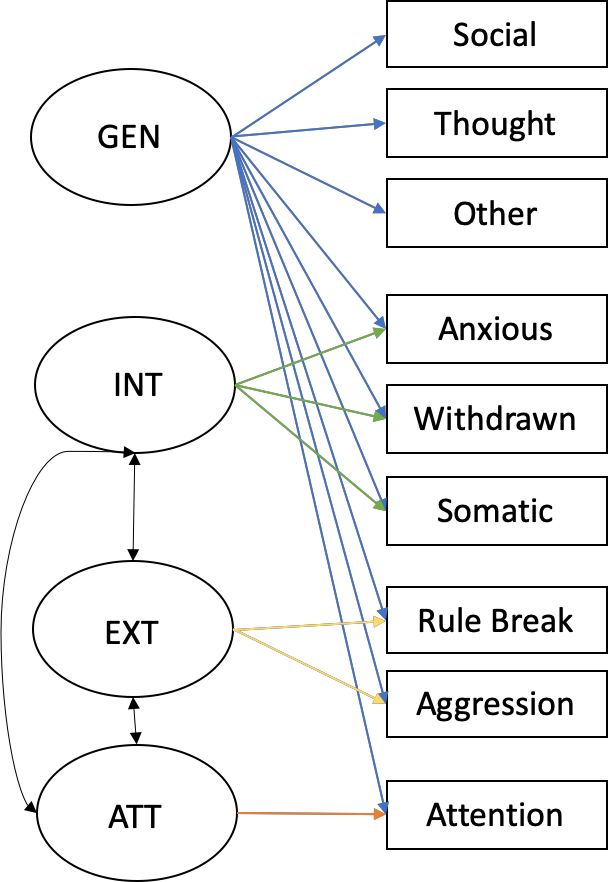


**Figure S2A: associations between cortical volume and internalizing/externalizing problem score adjusted for covariates, additionally adjusted for internalizing/externalizing problem score and total brain volume in ABCD study.**

**
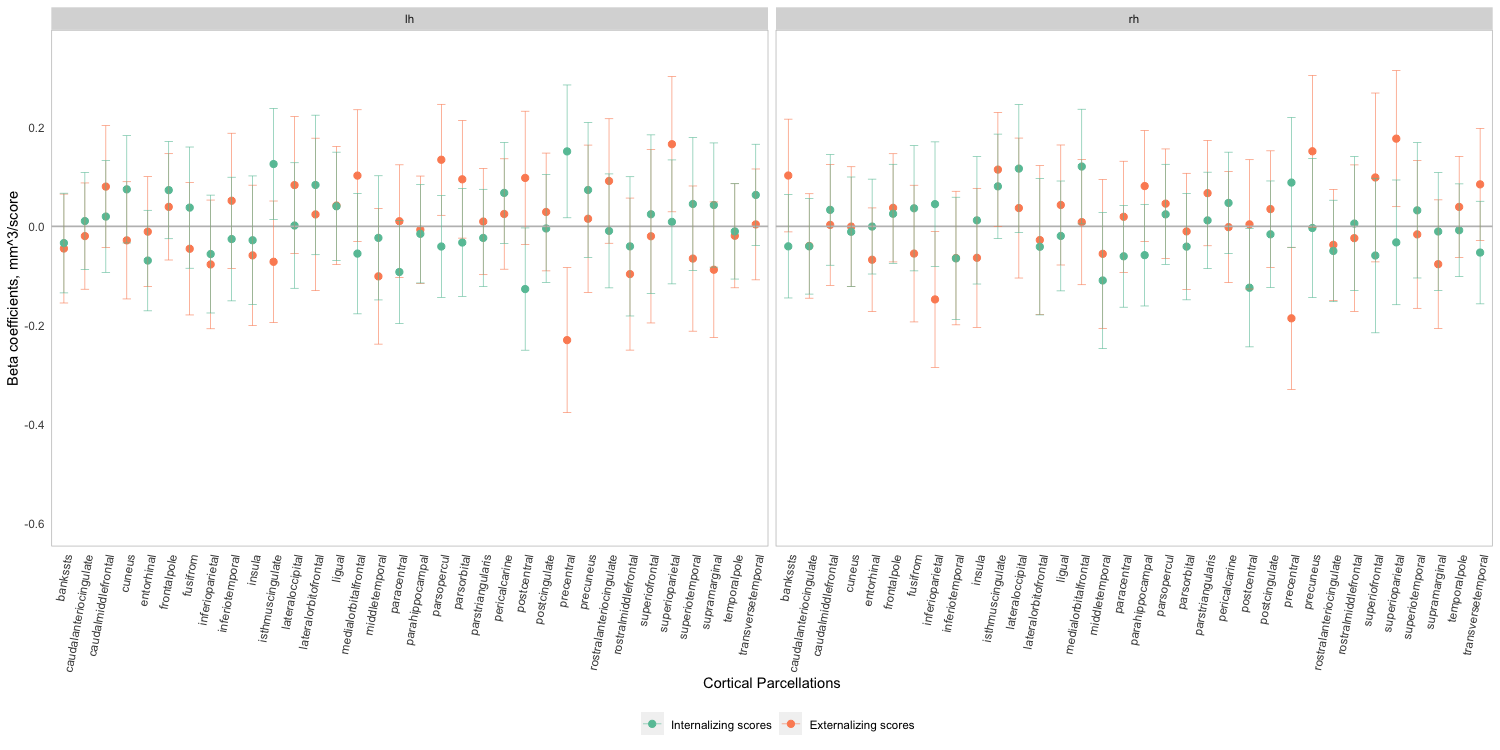
**

Note: The 95% confidence intervals shown in the figure represent nominal results without FDR correction.

For internalizing problems, 5 cortical regions were nominally significant, and none of them survived FDR correction. For externalizing problems, 6 regions were nominally significant, and none of them survived FDR correction.

**Figure S2B: associations between cortical volume and internalizing, externalizing and generalizing problem factor adjusted for covariates and total cortical volume (bifactor model) in ABCD study.**

**
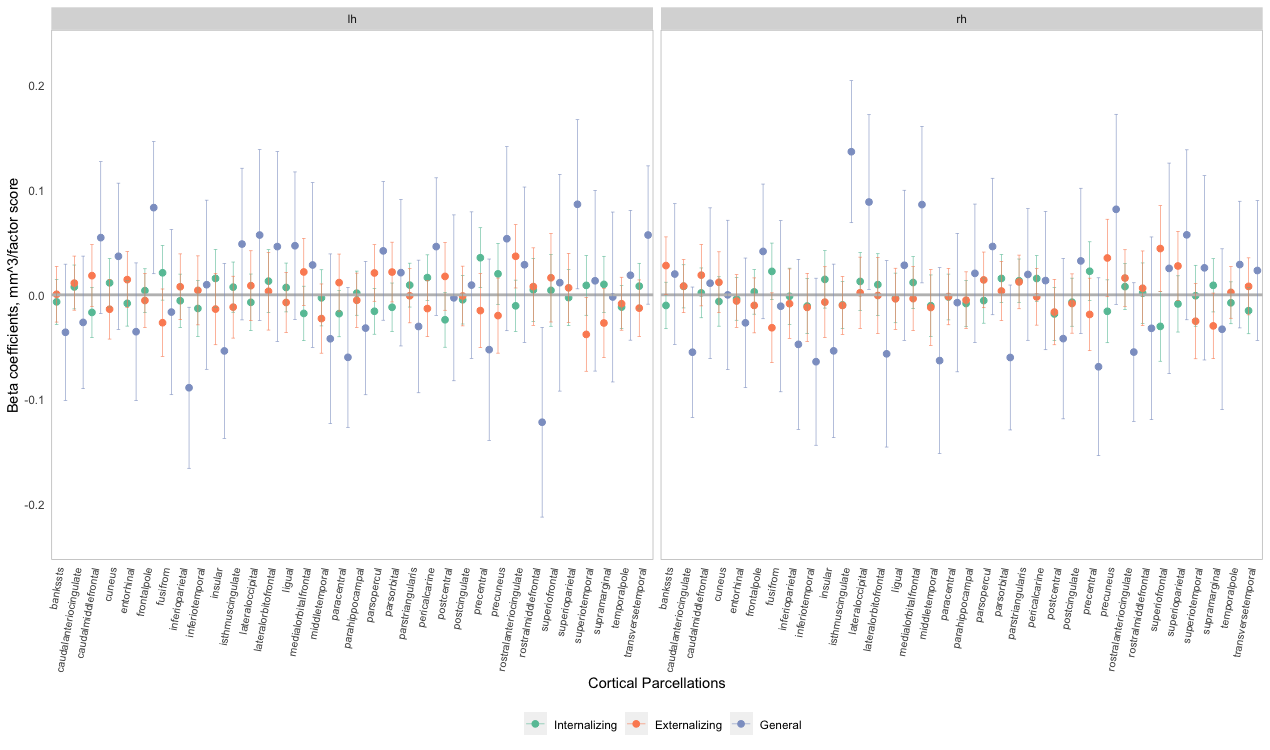
**

Note: The 95% confidence intervals shown in the figure represent nominal results without FDR correction.

For specific internalizing problems, precentral cortical volume in the left hemisphere was nominally significant, and it did not survive FDR correction. For specific externalizing problems, 4 cortical regions were nominally significant, and none of them survived FDR correction. For generalizing factor, 7 cortical regions were nominally significant, and only isthmus cingulate in the right hemisphere survived FDR correction.

**Figure S3A** associations between cortical volume and internalizing/externalizing problem score adjusted for covariates and mutually adjusted for internalizing/externalizing problem score, stratified by sex (boys)


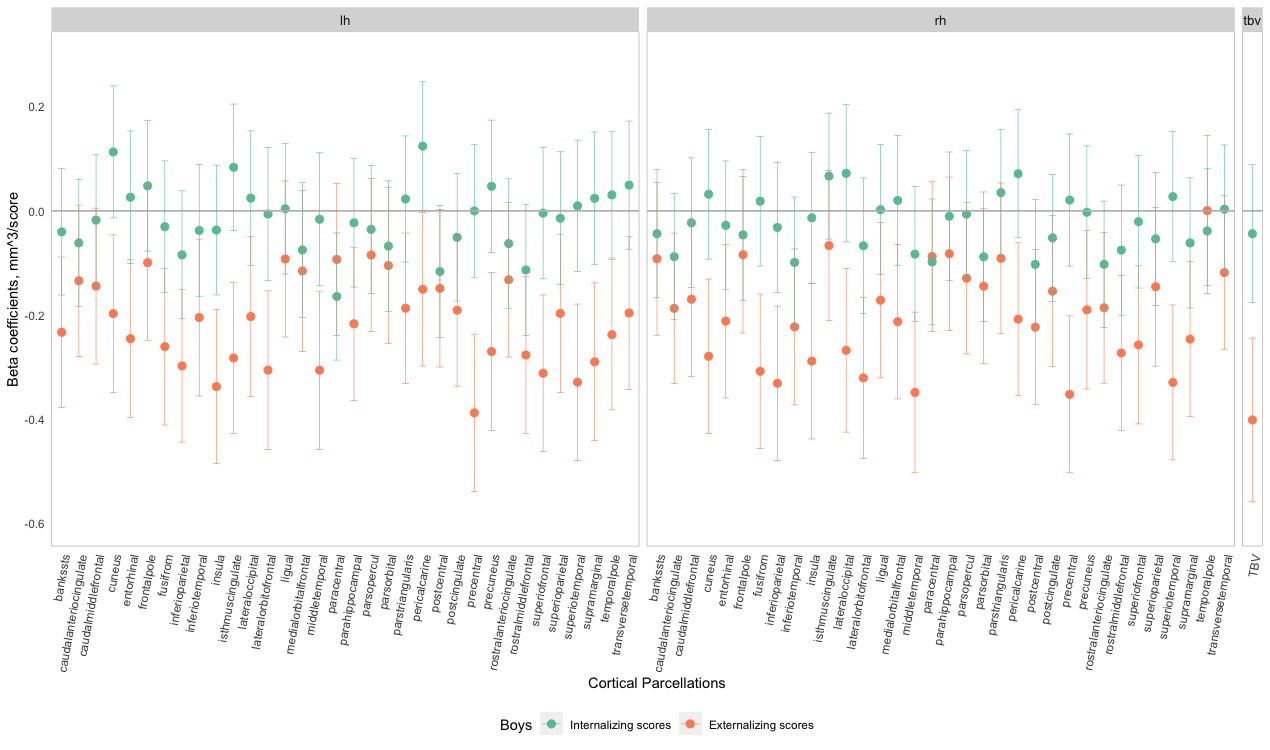


Note: The 95% confidence intervals shown in the figure represent nominal results without FDR correction.

For internalizing problems, 2 cortical regions were nominally significant, and none of them survived FDR correction. For externalizing problems, 47 cortical regions were nominally significant, and 45 of them survived FDR correction.

**Figure S3B** associations between cortical volume and internalizing/externalizing problem score adjusted for covariates and mutually adjusted for internalizing/externalizing problem score, stratified by sex (girls)


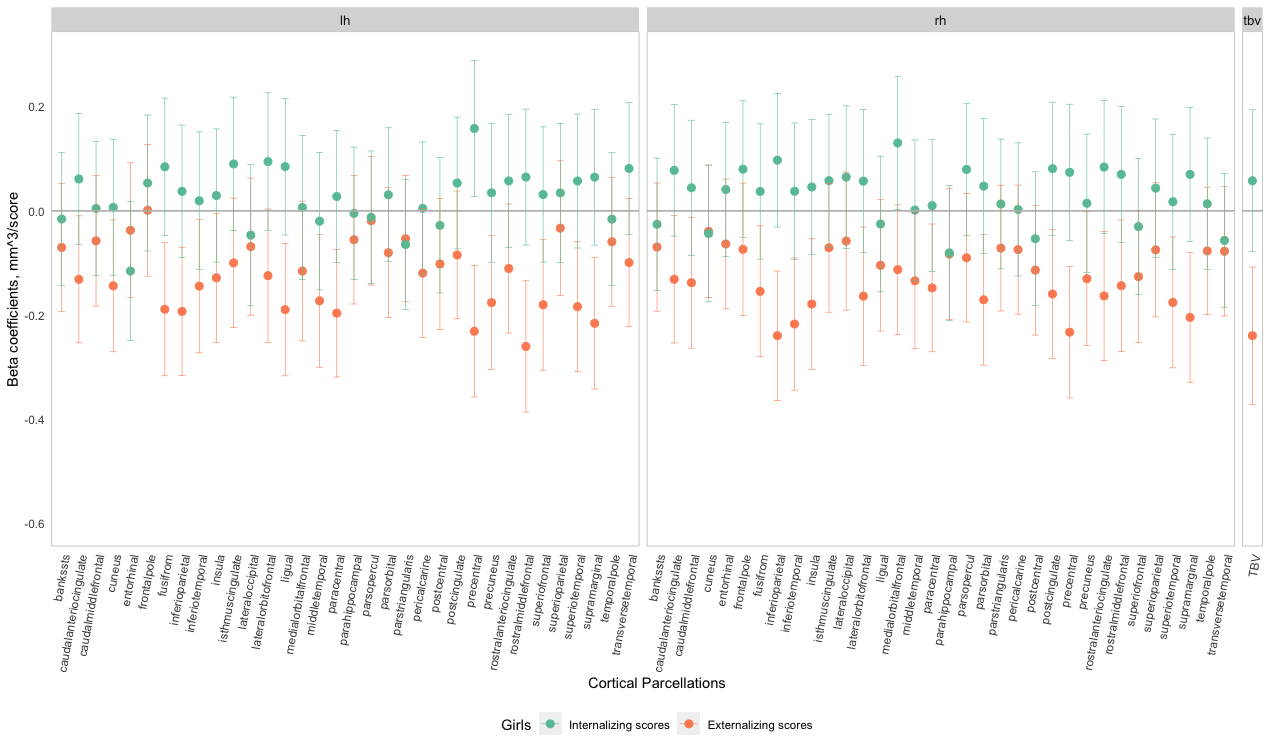


Note: The 95% confidence intervals shown in the figure represent nominal results without FDR correction.

For internalizing problems, 2 cortical regions were nominally significant, and none of them survived FDR correction. For externalizing problems, 32 cortical regions were nominally significant, and 23 of them survived FDR correction.

**Figure S4: associations between attention problem factor and cortical volumes (bifactor model)**

**
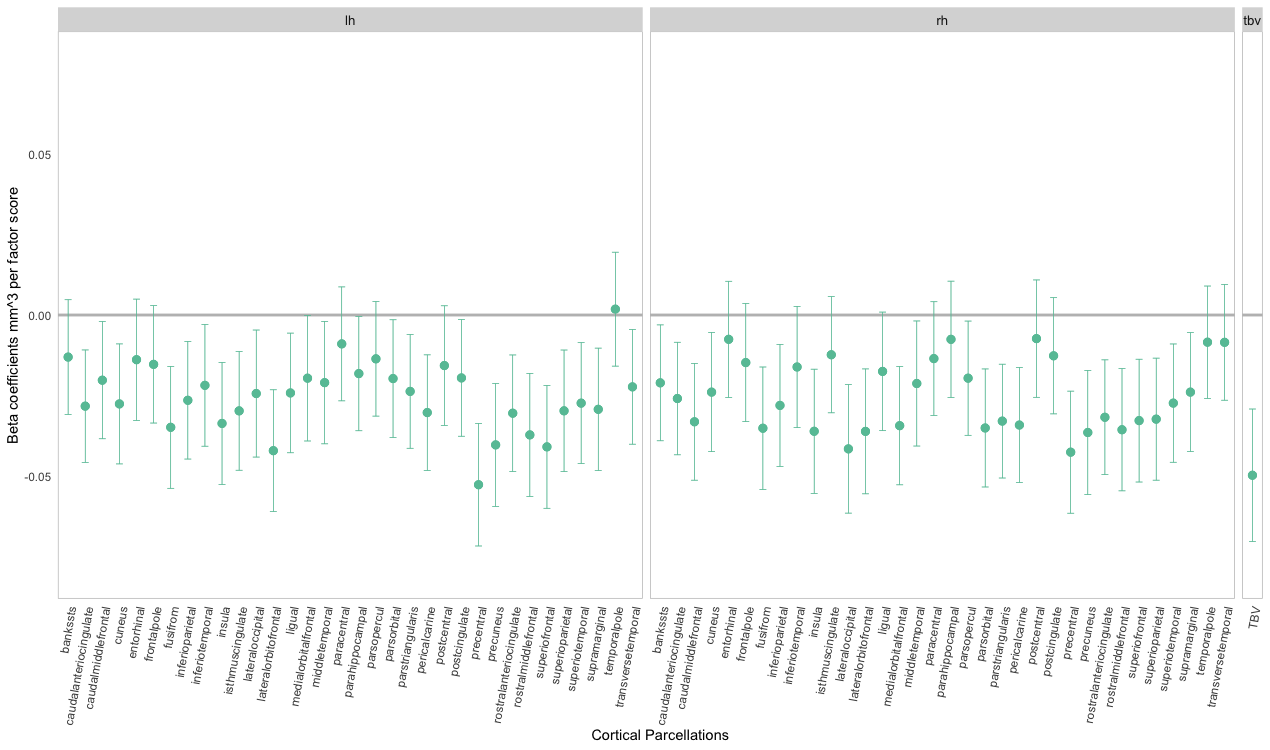
**

Note: The 95% confidence intervals shown in the figure represent nominal results without FDR correction.

For specific attention problems, 50 cortical regions were nominally significant, and 48 of them survived FDR correction.

**Figure S5: associations between cortical volume and internalizing/externalizing problem score adjusted for covariates in Generation R.**

**
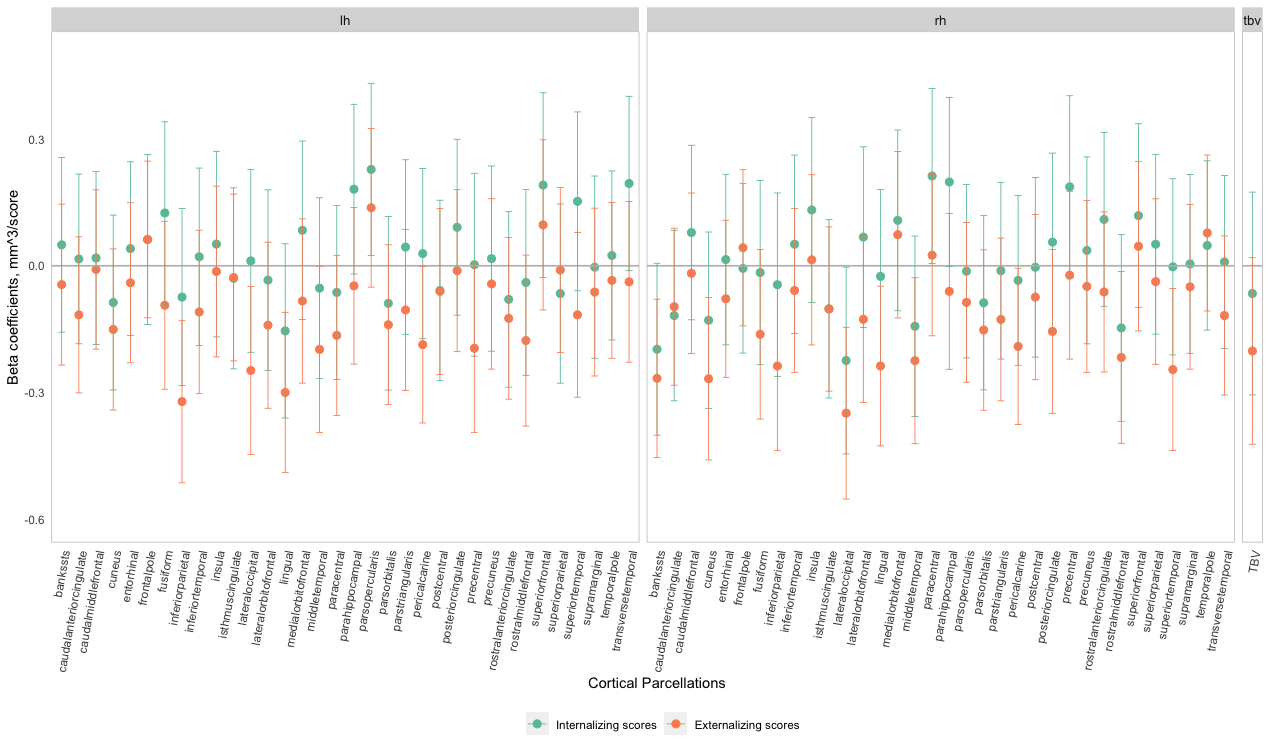
**

Note: The 95% confidence intervals shown in the figure represent nominal results without FDR correction.

For internalizing problems, 3 cortical regions were nominally significant, and none of them remained associated after FDR correction. For externalizing problems, 15 cortical regions were nominally significant, and 3 of them remained statistically significant after FDR correction.

**Figure S6A: associations between cortical volume and internalizing/externalizing problem score adjusted for covariates, mutually adjusted for internalizing/externalizing problem score and total brain volume in Generation R.**

**
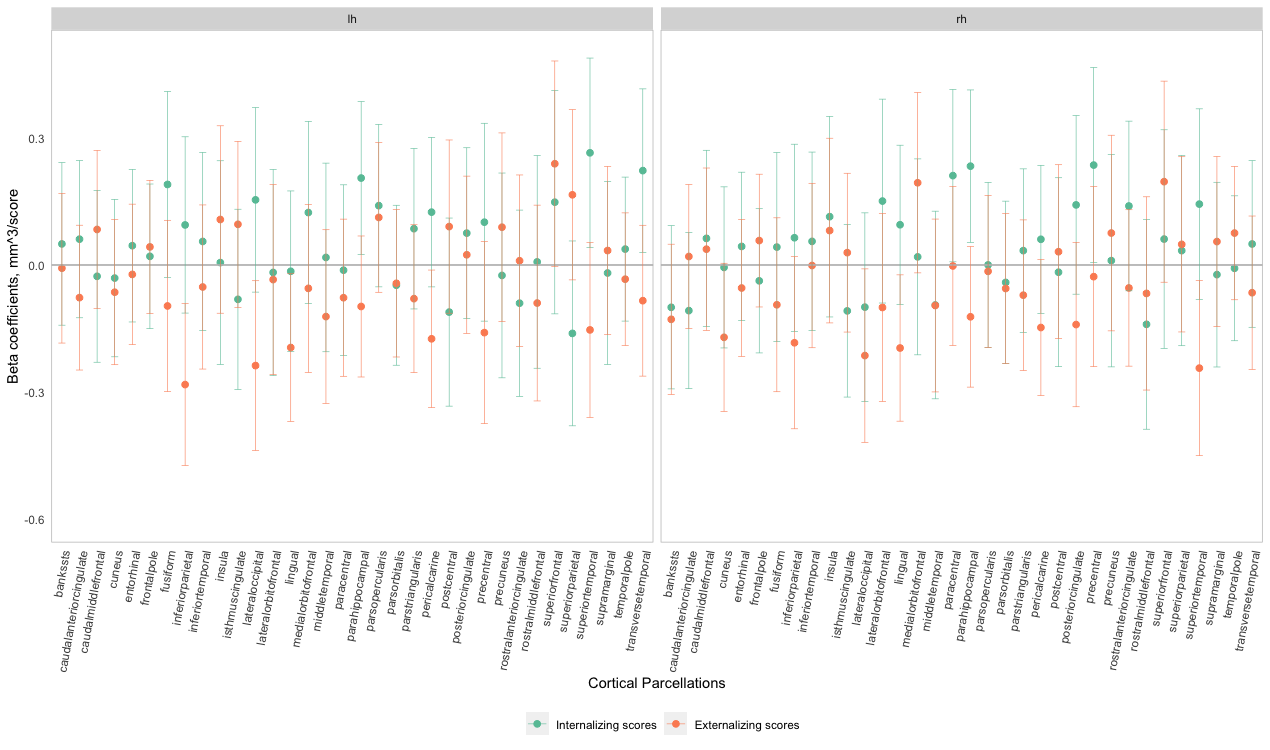
**

Note: The 95% confidence intervals shown in the figure represent nominal results without FDR correction.

For internalizing problems, 6 cortical regions were nominally significant, and none of them survived FDR correction. For externalizing problems, 12 regions were nominally significant, and only inferior parietal cortical volume in the left hemisphere remained statistically significant after FDR correction.

**Figure S6B: associations between cortical volume and internalizing, externalizing and generalizing problem factor adjusted for covariates and total brain volume (bifactor model) in Generation R.**

**
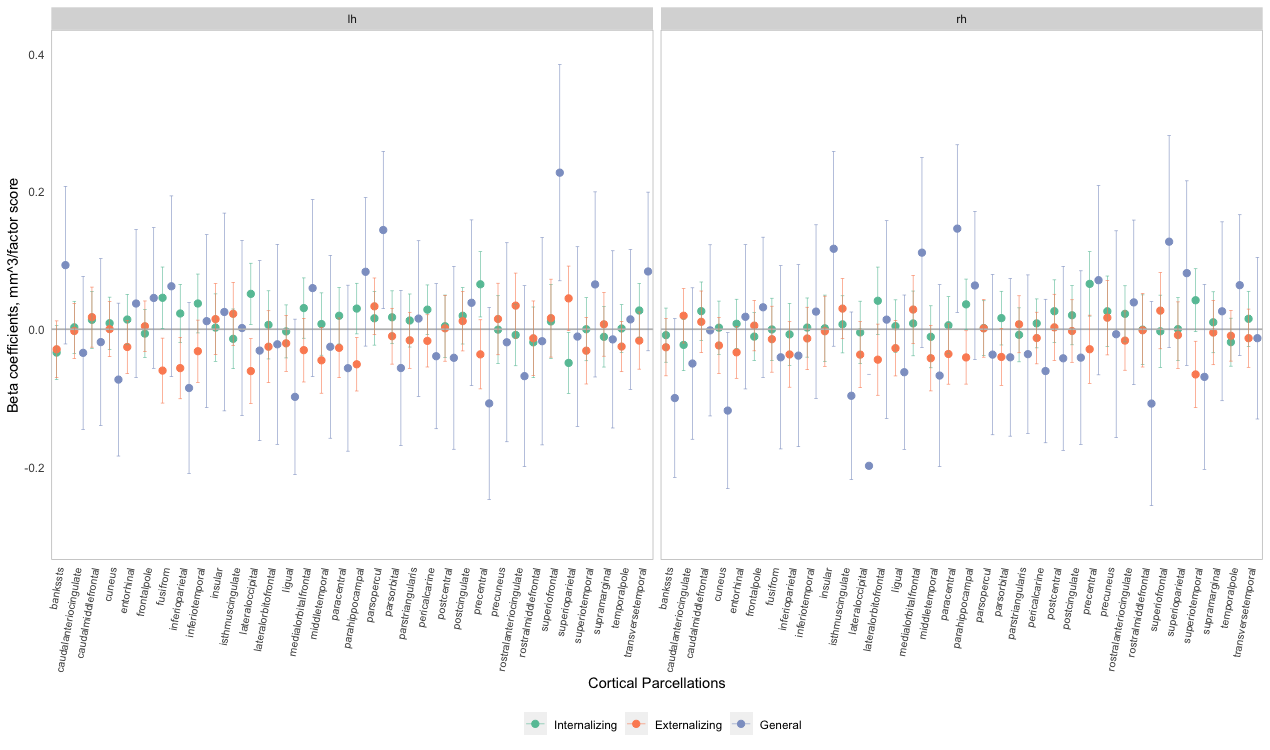
**

Note: The 95% confidence intervals shown in the figure represent nominal results without FDR correction.

For specific internalizing problems, 12 cortical regions were nominally significant, and none of them survived FDR correction. For specific externalizing problems, 10 cortical regions were nominally significant, and none of them survived FDR correction. For generalizing factor, 4 cortical regions were nominally significant, and only lateral occipital cortical volume in the right hemisphere survived FDR correction.

**Table S1 Cortical Regions abbreviation**

| **Cortical Regions** | **Abbreviation** |
| --- | --- |
| Banks of Superior Temporal Sulcus | bankssts |
| Caudal anterior cingulate | caudalanteriocingulate |
| Caudal middle frontal | caudalmiddlefrontal |
| Cuneus | cuneus |
| Entorhinal | entorhinal |
| Frontal pole | frontalpole |
| Fusiform | fusifrom |
| Inferior parietal | inferioparietal |
| Inferior temporal | inferiotemporal |
| Isthmus cingulate | isthmuscingulate |
| Insula | insula |
| Lateral occipital | lateraloccipital |
| Lateral orbitofrontal | lateralorbitofrontal |
| Lingual | ligual |
| Medial orbitofrontal | medialorbitalfrontal |
| Middle temporal | middletemporal |
| Para hippocampal | parahippocampal |
| Paracentral | paracentral |
| Pars opercularis | parsopercul |
| Pars orbitalis | parsorbital |
| Pars triangularis | parstriangularis |
| Pericalcarine | pericalcarine |
| Postcentral | postcentral |
| Posterior cingulate | postcingulate |
| Precentral | precentral |
| Precuneus | precuneus |
| Rostra lanterior cingulate | rostralanteriocingulate |
| Rostral middle frontal | rostralmiddlefrontal |
| Superior frontal | superiofrontal |
| Superior parietal | superioparietal |
| Superior temporal | superiotemporal |
| Supramarginal | supramarginal |
| Temporal pole | temporalpole |
| Transverse temporal | transversetemporal |

**Table S2 Comparison between included samples and excluded samples.**

|  | Included samples Mean±SD / N (%) | | Excluded samples Mean±SD / N (%) | P value* |
| --- | --- | --- | --- | --- |
| N | | 9635 | 2234 |  |
| Age (years) | | 9.91 ± 0.63 | 9.95 ± 0.61 | 0.001 |
| Sex | |  |  |  |
| Girls | | 4585 (47.6) | 1093 (48.9) | 0.26 |
| Boys | | 5050 (52.4) | 1141 (51.1) |  |
| Race | |  |  |  |
| non-Hispanic White | | 5746 (59.7) | 1653 (74.3) | <0.001 |
| non-Hispanic Black | | 1487 (15.5) | 384 (17.2) | 0.04 |
| Hispanic | | 2043 (21.2) | 106 (4.8) | <0.001 |
| Asian | | 216 (2.2) | 52 (2.3) | 0.87 |
| Native American/Pacific Islander | | 40 (0.4) | 12 (0.5) | 0.54 |
| other/unknown | | 87 (0.9) | 18 (0.8) | 0.75 |
| Family annual income | |  |  |  |
| Less than $50,000 | | 2688 (27.9) | 534 (23.9) | <0.001 |
| $50,000 - $100,000 | | 2497 (25.9) | 573 (25.6) | 0.82 |
| More than $100,000 | | 3618 (37.6) | 946 (42.3) | <0.001 |
| Missingness | | 832 (8.6) | 181 (8.1) | 0.44 |
| Parent education level (degree) | |  |  |  |
| High School graduation | | 1146 (11.9) | 238 (10.6) | 0.11 |
| Bachelor’s Degree | | 5200 (54.0) | 1218 (54.5) | 0.65 |
| Graduate Degree or above | | 3277 (34.0) | 766 (34.3) | 0.82 |
| Missingness | | 12 (0.1) | 12 (0.5) | <0.001 |
| Parental psychopathology (symptoms) | | 21.3 ± 18.1 | 20.6 ± 17.4 | 0.11 |
| Internalizing score (points) | | 5.1 ± 5.6 | 4.7 ± 5.3 | <0.001 |
| Externalizing score (points) | | 4.5 ± 5.9 | 4.3 ± 5.8 | 0.07 |
| Total CBCL score (points) | | 18.4 ± 18.1 | 17.4 ± 17.5 | 0.02 |

Note: Six individuals without CBCL data were excluded from the excluded samples.

*P values were derived from t-test for continuous variables and z-test for categorical variables.

Table S3 Associations with demographic covariates of psychiatric problems and total brain volume (N = 9635).

|  | Specific internalizing factor score,  Beta (95% CI) | Specific externalizing factor score,  Beta (95% CI) | General Psychopathology factor score,  Beta (95% CI) | Total brain volume, mm^3,  Beta (95% CI) |
| --- | --- | --- | --- | --- |
| Age, year | 0.024 (-0.005, 0.054) | -0.012 (-0.048, 0.024) | -0.06 (-0.17, 0.05) | -3062 (-4867, -1258) |
| Sex |  |  |  |  |
| Girls (vs Boys) | 0.32 (0.29, 0.36) | -0.21 (-0.26, 0.17) | -0.64 (0.77, -0.50) | -46987 (-49054, -44919) |
| Race (reference: non-Hispanic White) |  |  |  |  |
| non-Hispanic Black | -0.39 (-0.44, -0.34) | 0.47 (0.41, 0.54) | 0.23 (0.04, 0.42) | -52853 (-55891, -49814) |
| Hispanic | -0.006(-0.05, 0.04) | 0.02 (-0.03, 0.08) | 0.09 (-0.08, 0.26) | -26147 (-28836, -23456) |
| Asian | 0.024(-0.10, 0.15) | -0.01 (-0.16, 0.14) | -1.01 (-1.46, -0.55) | -26997 (-34235, -19758) |
| Native American/Pacific Islander | -0.19 (-0.48, 0.10) | 0.24 (-0.11, 0.59) | 0.31 (-0.73, 1.36) | -21899 (-38470, -5328) |
| other/unknown | -0.20 (-0.40, -0.01) | 0.22 (-0.02, 0.46) | 0.20 (-0.51, 0.91) | -29746 (-41027, -18464) |
| Family annual income  (reference: Less than $50,000) |  |  |  |  |
| $50,000 - $100,000 | 0.15 (0.10, 0.20) | -0.26 (-0.32, -0.20) | -0.70 (-0.88, -0.53) | 23446 (20450, 26443) |
| More than $100,000 | 0.18 (0.13, 0.22) | -0.31 (-0.37, -0.26) | -1.26 (-1.42, -1.09) | 34762 (32017, 37507) |
| Parent education level (degree)  (reference: High School graduation) |  |  |  |  |
| Bachelor’s Degree | 0.12 (0.06, 0.18) | -0.20 (-0.28, -0.13) | 0.13 (-0.08, 0.34) | 22530 (18981, 26079) |
| Graduate Degree or above | 0.25 (0.19, 0.31) | -0.39 (-0.47, -0.32) | -0.64 (-0.87, -0.42) | 39559 (35827, 43291) |
| Parental psychopathology (scores) | 0.002 (0001, 0.003) | 0.001 (-6e-05, 0.002) | 0.105 (0.102, 0.108) | -172 (-235, -110) |

**Table S4 Vertex-wise analysis results with covaried CBCL internalizing/externalizing problem scores and factor scores(bifactor model).**

| **Left Hemisphere** | **Cluster** | **n_vertices** | **mean_volume** | **mean_coefficient** | **top_region1** | **top_region2** | **top_region3** |
| --- | --- | --- | --- | --- | --- | --- | --- |
| Total internalizing score | 1 | 680 | 1.992 | 0.004 | fusiform (91.91%, 13.26%) | inferiortemporal (8.09%, 1.25%) | NA |
|  | 2 | 319 | 1.810 | 0.004 | lateraloccipital (100%, 5%) | NA | NA |
| Total externalizing score | 1 | 6225 | 1.961 | -0.004 | supramarginal (71.94%, 52.07%) | superiortemporal (22.94%, 19.64%) | transversetemporal (3.71%, 21.71%) |
|  | 2 | 5193 | 1.930 | -0.002 | insula (34.62%, 34.39%) | postcentral (23.51%, 12.83%) | superiortemporal (22.92%, 16.37%) |
|  | 3 | 3516 | 2.048 | -0.005 | middletemporal (70.42%, 55.62%) | bankssts (18.69%, 30.74%) | superiortemporal (7.25%, 3.51%) |
|  | 4 | 3757 | 2.006 | -0.003 | posteriorcingulate (44.34%, 51.01%) | precuneus (26.70%, 13.72%) | isthmuscingulate (23.00%, 34.14%) |
|  | 5 | 3252 | 1.895 | -0.003 | precentral (73.06%, 22.12%) | postcentral (26.94%, 9.20%) | NA |
|  | 6 | 2142 | 2.073 | -0.005 | inferiortemporal (62.75%, 30.44%) | fusiform (37.02%, 16.82%) | middletemporal (0.19%, 0.09%) |
|  | 7 | 1628 | 2.033 | -0.004 | inferiorparietal (83.91%, 17.35%) | superiorparietal (16.09%, 2.51%) | NA |
|  | 8 | 1916 | 2.076 | -0.004 | lateralorbitofrontal (80.95%, 37.03%) | insula (19.05%, 6.98%) | NA |
|  | 9 | 746 | 1.918 | -0.004 | rostralmiddlefrontal (79.36%, 8.17%) | superiorfrontal (8.18%, 0.50%) | frontalpole (6.43%, 17.65%) |
|  | 10 | 605 | 2.117 | -0.004 | superiorfrontal (91.57%, 4.55%) | rostralmiddlefrontal (8.43%, 0.70%) | NA |
|  | 11 | 496 | 1.742 | -0.005 | superiorfrontal (89.11%, 3.63%) | rostralanteriorcingulate (10.89%, 4.00%) | NA |
|  | 12 | 535 | 1.964 | -0.003 | fusiform (100%, 11.35%) | NA | NA |
|  | 13 | 587 | 1.920 | -0.005 | inferiorparietal (100%, 7.46%) | NA | NA |
|  | 14 | 298 | 1.852 | -0.005 | inferiorparietal (68.79%, 2.60%) | lateraloccipital (31.21%, 1.46%) | NA |
| **Right Hemisphere** | **Cluster** | **n_vertices** | **mean_volume** | **mean_coefficient** | **top_region1** | **top_region2** | **top_region3** |
| Total internalizing score | 1 | 753 | 1.922 | 0.005 | lateraloccipital (100%, 12.63%) | NA | NA |
| Total externalizing score | 1 | 10382 | 1.953 | -0.005 | inferiorparietal (25.61%, 27.48%) | middletemporal (22.09%, 45.34%) | fusiform (20.08%, 44.73%) |
|  | 2 | 4733 | 1.984 | -0.003 | supramarginal (52.25%, 30.34%) | superiortemporal (38.62%, 26.62%) | bankssts (4.50%, 9.70%) |
|  | 3 | 4602 | 1.915 | -0.003 | precentral (79.94%, 34.37%) | postcentral (20.06%, 10.10%) | NA |
|  | 4 | 3384 | 2.054 | -0.002 | insula (47.13%, 31.34%) | postcentral (28.31%, 10.48%) | precentral (24.56%, 7.76%) |
|  | 5 | 1788 | 1.980 | -0.003 | precuneus (84.34%, 18.91%) | cuneus (11.13%, 12.15%) | isthmuscingulate (4.53%, 3.39%) |
|  | 6 | 2168 | 2.107 | -0.003 | posteriorcingulate (67.57%, 48.93%) | superiorfrontal (13.84%, 2.53%) | paracentral (7.56%, 4.28%) |
|  | 7 | 1668 | 2.095 | -0.003 | lateralorbitofrontal (69.24%, 26.53%) | insula (29.44%, 9.65%) | superiortemporal (1.32%, 0.32%) |
|  | 8 | 790 | 1.839 | -0.005 | middletemporal (67.97%, 10.62%) | bankssts (32.03%, 11.52%) | NA |
|  | 9 | 730 | 2.263 | -0.004 | supramarginal (93.01%, 8.33%) | postcentral (6.99%, 0.56%) | NA |
|  | 10 | 395 | 1.829 | -0.003 | lateralorbitofrontal (100%, 9.07%) | NA | NA |
|  | 11 | 319 | 2.209 | -0.004 | parsorbitalis (73.04%, 24.63%) | lateralorbitofrontal (26.96%, 1.98%) | NA |
| **Left Hemisphere** | **Cluster** | **n_vertices** | **mean_volume** | **mean_coefficient** | **top_region1** | **top_region2** | **top_region3** |
| Specific Internalizing scores | 1 | 8317 | 2.005 | 0.023 | lateraloccipital (37.50%, 48.89%) | fusiform (35.77%, 63.11%) | inferiortemporal (19.74%, 37.19%) |
|  | 2 | 7447 | 2.027 | 0.018 | lateralorbitofrontal (35.18%, 62.56%) | insula (29.92%, 42.61%) | superiortemporal (15.19%, 15.55%) |
|  | 3 | 8075 | 1.995 | 0.016 | precuneus (53.63%, 59.26%) | posteriorcingulate (19.44%, 48.07%) | cuneus (11.00%, 54.48%) |
|  | 4 | 8212 | 1.918 | 0.017 | supramarginal (43.81%, 41.84%) | superiortemporal (20.56%, 23.22%) | postcentral (17.44%, 15.04%) |
|  | 5 | 1792 | 1.986 | 0.020 | lingual (87.5%, 37.29%) | parahippocampal (12.5%, 12.19%) | NA |
|  | 6 | 2724 | 1.907 | 0.012 | precentral (90.97%, 23.07%) | postcentral (9.03%, 2.58%) | NA |
|  | 7 | 1566 | 2.022 | 0.027 | middletemporal (84.99%, 29.90%) | superiortemporal (12.58%, 2.71%) | inferiortemporal (2.43%, 0.86%) |
|  | 8 | 1266 | 2.085 | 0.020 | superiorfrontal (100%, 10.39%) | NA | NA |
|  | 9 | 1348 | 2.081 | 0.022 | superiorparietal (100%, 12.89%) | NA | NA |
|  | 10 | 804 | 2.070 | 0.017 | precentral (54.60%, 4.09%) | parsopercularis (33.08%, 8.53%) | caudalmiddlefrontal (12.31%, 2.65%) |
|  | 11 | 395 | 1.774 | 0.022 | superiorfrontal (100%, 3.24%) | NA | NA |
|  | 12 | 581 | 1.859 | 0.013 | inferiorparietal (68.67%, 5.07%) | superiorparietal (31.33%, 1.74%) | NA |
|  | 13 | 460 | 2.081 | 0.012 | parstriangularis (99.35%, 22.34%) | parsopercularis (0.65%, 0.10%) | NA |
| Specific Externalizing score | 1 | 1978 | 2.001 | -0.013 | supramarginal (96.36%, 22.16%) | postcentral (2.53%, 0.53%) | superiortemporal (1.11%, 0.30%) |
|  | 2 | 1460 | 1.984 | -0.022 | middletemporal (89.04%, 29.20%) | inferiortemporal (5.75%, 1.90%) | superiortemporal (5.21%, 1.05%) |
|  | 3 | 1043 | 2.114 | -0.013 | lateralorbitofrontal (81.88%, 20.39%) | insula (18.12%, 3.61%) | NA |
|  | 4 | 671 | 1.987 | -0.016 | inferiortemporal (54.25%, 8.24%) | fusiform (45.75%, 6.51%) | NA |
|  | 5 | 684 | 2.113 | -0.012 | precuneus (53.36%, 4.99%) | isthmuscingulate (46.64%, 12.60%) | NA |
|  | 6 | 461 | 1.899 | -0.014 | fusiform (100%, 9.78%) | NA | NA |
|  | 7 | 462 | 1.993 | -0.010 | precentral (100%, 4.3%) | NA | NA |
|  | 8 | 350 | 1.782 | -0.013 | precuneus (100%, 4.79%) | NA | NA |
| **Right Hemisphere** | **Cluster** | **n_vertices** | **mean_volume** | **mean_coefficient** | **top_region1** | **top_region2** | **top_region3** |
| Specific Internalizing scores | 1 | 8521 | 2.015 | 0.016 | lateralorbitofrontal (30.41%, 59.51%) | insula (29.43%, 49.27%) | rostralmiddlefrontal (12.39%, 13.43%) |
|  | 2 | 7057 | 2.001 | 0.017 | precuneus (47.31%, 41.87%) | cuneus (12.95%, 55.80%) | posteriorcingulate (10.26%, 24.18%) |
|  | 3 | 6149 | 1.886 | 0.026 | fusiform (49.29%, 65.03%) | middletemporal (27.66%, 33.64%) | inferiortemporal (19.91%, 29.16%) |
|  | 4 | 3791 | 1.991 | 0.029 | lateraloccipital (80.82%, 51.38%) | inferiorparietal (15.14%, 5.93%) | fusiform (3.72%, 3.03%) |
|  | 5 | 3558 | 1.975 | 0.014 | precentral (100%, 33.24%) | NA | NA |
|  | 6 | 1363 | 2.025 | 0.017 | supramarginal (46.96%, 7.85%) | superiortemporal (39.18%, 7.78%) | bankssts (13.87%, 8.61%) |
|  | 7 | 768 | 1.901 | 0.018 | lingual (76.17%, 15.02%) | parahippocampal (23.83%, 10.51%) | NA |
|  | 8 | 927 | 1.809 | 0.022 | superiorparietal (83.28%, 7.55%) | inferiorparietal (16.72%, 1.60%) | NA |
|  | 9 | 808 | 2.012 | 0.015 | superiorparietal (91.21%, 7.21%) | postcentral (8.79%, 0.78%) | NA |
|  | 10 | 739 | 2.072 | 0.015 | superiortemporal (84.71%, 9.11%) | transversetemporal (14.88%, 14.08%) | supramarginal (0.41%, 0.04%) |
|  | 11 | 740 | 1.798 | 0.015 | supramarginal (64.32%, 5.84%) | postcentral (35.68%, 2.89%) | NA |
|  | 12 | 805 | 1.778 | 0.014 | precuneus (83.48%, 8.43%) | superiorparietal (15.78%, 1.24%) | postcentral (0.75%, 0.07%) |
|  | 13 | 463 | 1.999 | 0.012 | precentral (100%, 4.33%) | NA | NA |
| Specific Externalizing scores | 1 | 1141 | 1.997 | -0.021 | lateraloccipital (75.46%, 14.44%) | inferiorparietal (19.46%, 2.29%) | inferiortemporal (4.82%, 1.31%) |
|  | 2 | 850 | 1.782 | -0.020 | middletemporal (97.76%, 16.43%) | superiortemporal (1.65%, 0.20%) | inferiortemporal (0.59%, 0.12%) |
|  | 3 | 702 | 1.909 | -0.017 | fusiform (97.29%, 14.65%) | lingual (2.71%, 0.49%) | NA |
|  | 4 | 684 | 1.942 | -0.016 | fusiform (57.31%, 8.41%) | inferiortemporal (42.69%, 6.96%) | NA |
|  | 5 | 735 | 2.051 | -0.013 | posteriorcingulate (69.12%, 16.97%) | isthmuscingulate (30.88%, 9.51%) | NA |
|  | 6 | 610 | 1.737 | -0.007 | precentral (99.02%, 5.64%) | postcentral (0.98%, 0.07%) | NA |
|  | 7 | 516 | 2.004 | -0.012 | superiortemporal (94.57%, 7.11%) | transversetemporal (5.43%, 3.59%) | NA |
|  | 8 | 548 | 1.848 | -0.012 | supramarginal (82.85%, 5.57%) | postcentral (17.15%, 1.03%) | NA |
|  | 9 | 470 | 2.154 | -0.013 | lateralorbitofrontal (88.09%, 9.51%) | insula (11.91%, 1.10%) | NA |

**Table S5 Demographic distribution of GenR**

|  | **Mean±SD / N (%)** | |
| --- | --- | --- |
| N | | 2365 |
| Age (years) | | 10.11 ± 0.57 |
| Sex | |  |
| Girls | | 1206 (51.0%) |
| Boys | | 1159 (49.0%) |
| Ethnicity | |  |
| Dutch | | 1529 (64.7%) |
| Non-Dutch western | | 218 (9.2%) |
| Non-western | | 594 (25.1%) |
| Missingness | | 24 (1.0%) |
| Family monthly income (median) | | €3200 - €4000 |
| Low income (< €2000/month) | | 351 (14.8%) |
| Medium income (€2000 - €4000/month) | | 911 (38.5%) |
| High income (> €4000/month) | | 825 (34.9%) |
| Missingness | | 278 (11.8%) |
| Maternal education | |  |
| Low | | 180 (7.6%) |
| Medium | | 604 (25.5%) |
| High | | 1395 (59.0%) |
| Missingness | | 186 (7.9%) |
| Maternal psychopathology (GSI) | | 0.30 ± 0.39 |
| Missingness | | 709 (30.0%) |
| Internalizing scores | | 4.75 ± 4.96 |
| Externalizing scores | | 3.75 ± 4.59 |
| Total CBCL scores | | 17.17 ± 15.05 |

**Table S6 Intraclass correlations (ICC) of the coefficients in ABCD and Generation R studies for 68 cortical volumes and total volumes**

|  | Outcome | ICC | ICC 95% CI |
| --- | --- | --- | --- |
| CBCL original scores adjusted for covariates | Internalizing problems | 0.955 | (0.929, 0.972) |
|  | Externalizing problems | 0.994 | (0.99, 0.996) |
| CBCL original scores adjusted for covariates and INT/EXT | Internalizing problems | 0.946 | (0.914, 0.966) |
|  | Externalizing problems | 0.995 | (0.992, 0.997) |
| CBCL original scores adjusted for covariates, INT/EXT and TBV | Internalizing problems | 0.993 | (0.989, 0.996) |
|  | Externalizing problems | 0.969 | (0.950, 0.981) |
| CBCL bifactor scores adjusted for covariates | Specific internalizing problems | 0.881 | (0.814, 0.924) |
|  | Specific externalizing problems | 0.971 | (0.953, 0.982) |
|  | General psychopathology | 0.947 | (0.916, 0.967) |
| CBCL bifactor scores adjusted for covariates and TBV | Specific internalizing problems | 0.901 | (0.844, 0.938) |
|  | Specific externalizing problems | 0.958 | (0.932, 0.974) |
|  | General psychopathology | 0.98 | (0.967, 0.987) |
